# Naringin Inhibits Apoptosis Induced by Cyclic Stretch in Rat Annular Cells and Partially Attenuates Disc Degeneration by Inhibiting the ROS/NF-*κ*B Pathway

**DOI:** 10.1155/2022/6179444

**Published:** 2022-02-23

**Authors:** Yue-Hui Zhang, Wen-Ji Shangguan, Zhi-Jun Zhao, Fu-Chao Zhou, Hai-Tao Liu, Zhi-Hui Liang, Jia Song, Jiang Shao

**Affiliations:** ^1^Spine Center, Xin Hua Hospital Affiliated to Shanghai Jiao Tong University School of Medicine, Shanghai 200092, China; ^2^Department of Traditional Chinese Medicine, Renji Hospital, School of Medicine, Shanghai Jiaotong University, Shanghai 200127, China; ^3^Department of Orthopedic Surgery, Yangzhong Traditional Chinese Medicine Hospital, Yangzhong 212200, China

## Abstract

Oxidative stress and apoptosis play important roles in the pathogenesis of various degenerative diseases. Previous studies have shown that naringin can exert therapeutic effects in multiple degenerative diseases by resisting oxidative stress and inhibiting apoptosis. Although naringin is effective in treating degenerative disc disease, the underlying mechanism remains unclear. This study is aimed at investigating the effects of naringin on oxidative stress, apoptosis, and intervertebral disc degeneration (IVDD) induced by cyclic stretch and the underlying mechanisms *in vitro* and *in vivo*. Abnormal cyclic stretch was applied to rat annulus fibrosus cells, which were then treated with naringin, to observe the effects of naringin on apoptosis, oxidative stress, mitochondrial function, and the nuclear factor- (NF-) *κ*B signaling pathway. Subsequently, a rat model of IVDD induced by dynamic and static imbalance was established to evaluate the effects of naringin on the degree of degeneration (using imaging and histology), apoptosis, and oxidative stress in the serum and the intervertebral disc. Naringin inhibited the cyclic stretch-induced apoptosis of annulus fibrosus cells, reduced oxidative stress, improved mitochondrial function, enhanced the antioxidant capacity, and suppressed the activation of the NF-*κ*B signaling pathway. Additionally, it reduced the degree of IVDD (evaluated using magnetic resonance imaging) and the level of oxidative stress and inhibited apoptosis and p-P65 expression in the intervertebral discs of rats. Thus, naringin can inhibit cyclic stretch-induced apoptosis and delay IVDD, and the underlying mechanism may be related to the inhibition of oxidative stress and activation of the NF-*κ*B signaling pathway. Naringin may be an effective drug for treating degenerative disc disease.

## 1. Introduction

Intervertebral disc degeneration- (IVDD-) related diseases can significantly affect the mobility and quality of life of patients, thereby imposing a burden on the individual as well as the society [1, 2]. The prevalence of IVDD-related diseases is increasing, and the age of onset is gradually decreasing [3]. The etiology of IVDD is complex and multifactorial, and its molecular mechanism is currently unclear. Studies have shown that the apoptosis of intervertebral disc cells owing to excessive stress stimulation reduces the number of active cells in the intervertebral discs and subsequently reduces the synthesis and alters the composition of the extracellular matrix (ECM); this is an important cause of IVDD [3, 4]. Moreover, we previously found that the mitochondrial and endoplasmic reticulum-mediated pathways of apoptosis are involved in rat annulus fibrosus (AF) cell apoptosis induced by cyclic stretch; however, apoptosis could not be completely prevented by inhibiting these two pathways [5, 6]. This indicated the complexity of the mechanism underlying the cyclic stretch-induced apoptosis of intervertebral disc cells.

The free radical theory of aging suggests that declining tissue and organ functions are closely related to oxidative stress induced by reactive oxygen species (ROS) [7]. Intracellular redox homeostasis depends on the balance between ROS generation and ROS scavenging by antioxidants, such as superoxide dismutase (SOD), glutathione peroxidase (GPx), glutathione (GSH), and catalase (CAT), among others. Disturbances in this balance are detrimental to the fate of cells. Excess ROS cause oxidative damage to DNA, lipids, and proteins, and the by-products of oxidative stress, such as malondialdehyde (MDA), a product of the peroxidation of polyunsaturated fatty acid residues, accumulate concomitantly [7, 8]. ROS and oxidative stress exert pathogenic effects in various bone and muscle diseases, primarily by regulating the nuclear factor kappa beta (NF-*κ*B) pathway, mitogen-activated protein kinase pathway, and phosphatidylinositol 3-kinase pathway [7, 9–12]. Normal intervertebral disc tissues are characterized by a hypoxic microenvironment resulting from the lack of blood vessels. Previously, oxidative stress was not considered to be associated with IVDD. However, multiple recent studies have confirmed that intervertebral disc cells metabolize oxygen *in vivo* and produce ROS in the microenvironment of intervertebral discs, thereby serving as an important medium for regulating ECM metabolism, proinflammatory factor phenotypes, apoptosis, autophagy, and aging [8, 13–15].

Naringin, a monomer extracted from the total flavonoids of *Rhizoma Drynariae*, exerts various biological effects, including antioxidant, angiogenic, and anti-inflammatory effects [16]. The antioxidant effect of naringin, which can target various organs and presents a potential therapeutic value for several degenerative diseases, has been considered a research hotspot in recent times [17]. The protective effect of naringin was shown to be primarily mediated through modulations of various enzymatic and nonenzymatic antioxidant parameters (increased GSH, GPx, SOD, and CAT and decreased MDA) in various organs [17, 18]. *Rhizoma Drynariae*, a traditional Chinese medicine, is commonly used to treat degenerative disc diseases [19] and osteoporosis [18, 20] in China; however, the mechanism underlying its action remains unclear. Previous studies have shown that naringin can inhibit the oxidative stress-induced apoptosis of nucleus pulposus cells and mesenchymal stem cells, suppress the release of inflammatory mediators, regulate ECM metabolism in intervertebral discs, and delay IVDD [21–24]. Additionally, it was confirmed that naringin can inhibit the release of pain mediators, relieve discogenic low back pain, and suppress IVDD [19, 21]. Therefore, naringin could be a suitable candidate for the treatment of IVDD-related diseases. However, the effect of naringin on cyclic stretch-induced apoptosis in intervertebral disc cells remains unclear. In this study, the effects of naringin on oxidative stress, apoptosis, and IVDD induced by cyclic stretch and the underlying mechanisms i*n vitro* and *in vivo* were investigated to provide a theoretical basis for the application of naringin in IVDD treatment.

## 2. Materials and Methods

### 2.1. *In Vitro* Experiment

#### 2.1.1. Isolation and Culture of Rat AF Cells

The study was approved by the institutional ethics committee (Approval No. XHEC-D-2020-172). Eight 3-month-old male Sprague-Dawley (SD) rats were euthanized by intraperitoneal overdose of pentobarbital sodium (150 mg/kg). Under aseptic conditions, the whole lumbar spine was removed, the L1–L6 intervertebral discs were separated, ligaments surrounding the intervertebral discs were removed, and the nucleus pulposus was scraped using a small curet. Subsequently, the AF, removed from five lumbar intervertebral discs of one rat, was collected in a 6 cm dish, washed with Hanks' balanced salt solution (HBSS; Gibco, Grand Island, NY, USA), cut into fragments of approximately 1 mm^3^, transferred to a 15 mL centrifuge tube, digested with 0.4% pronase (Roche, Basel, Switzerland) for 90 min with shaking once every 15 min, and digested overnight with 0.025% type II collagenase and 0.01% type V hyaluronidase (Sigma-Aldrich, St. Louis, MO, USA) in a shaker at 37°C (110 rpm). Tissue fragments were removed using a cell strainer with a pore size of 70 *μ*m. Next, the separated cells were washed three times with HBSS, resuspended in a complete culture medium (Dulbecco's Modified Eagle Medium/F-12 containing 10% fetal bovine serum, 100 U/mL penicillin, and 100 *μ*g/mL streptomycin; Gibco), seeded in a 6 cm dish, and cultured in a cell incubator with 5% CO_2_ at 37°C; the medium was refreshed every day. After the cells reached 90% confluence, they were dissociated using 0.05% trypsin–ethylenediaminetetraacetic acid (EDTA) and replated into appropriate culture plates after washing with HBSS. All cells used in the study were first-passage AF cells maintained in a monolayer, and all experiments were performed in triplicate (*n* = 3). Naringin was reconstituted in dimethyl sulfoxide (DMSO) to prepare a stock solution of 500 *μ*g/mL, and a 0 *μ*g/mL naringin group was used as the *in vitro* vehicle group.

#### 2.1.2. Cellular Activity Test

Rat AF cells were seeded in 96-well plates at 2 × 10^4^ cells per well; incubated overnight; treated with complete medium containing 0, 1, 10, 50, 100, or 200 *μ*g/mL naringin (Sigma-Aldrich); and subsequently cultured for 48 h. Next, 0.5 mg/mL MTT solution (C0009S Beyotime, Shanghai, China) was added to each well, and the cells were cultured at 37°C for another 4 h. Subsequently, the liquid in the culture plate was removed, and 100 *μ*L of DMSO (Sigma-Aldrich) was added to dissolve the blue-purple crystals formed by MTT, followed by shaking for 10 min. The optical densities (OD) in the control and treatment groups were then measured at 570 nm using an automatic microplate reader (BioTek, Winooski, VT, USA).

#### 2.1.3. Application of Cyclic Stretch

Rat AF cells were seeded in BioFlex 6-well plates (Flexcell International, Hillsborough, NC, USA) at 2 × 10^5^ cells/well. When the cells reached 80–90% confluence, the culture medium was replaced with DMEM/F-12 containing 1% fetal bovine serum, and the cells were incubated for another 24 h to allow synchronization. Once the culture medium was replaced with 2 mL of complete medium, naringin was added at different concentrations, and the cells were then incubated for 2 h, after which the cells were stretched using the Flexcell Tension Plus system (FX-5000T, Flexcell International). The stretch parameters were 0.5 Hz and 20% stretch deformation, as described in our previous study [5]. The cells were collected for subsequent experiments after 36 h of stretching. The groups in the *in vitro* experiments were as follows: control group: static cells, used as negative controls; stretch group: cells stretched without naringin treatment (0 *μ*g/mL), used as positive controls; and naringin treatment groups: cells stretched and treated with naringin at a low (50 *μ*g/mL) or high (100 *μ*g/mL) concentration.

#### 2.1.4. Hoechst 33258 Staining

Cells were seeded in BioFlex 6-well plates at 2 × 10^5^ cells/well. After treatment under the aforementioned conditions for 36 h, morphological changes were observed under an inverted phase contrast microscope (Olympus, Tokyo, Japan). Next, the culture medium was removed by pipetting, and 2 *μ*g/mL Hoechst 33258 (C0003, Beyotime, Shanghai, China) was added. Morphological changes in the nuclei were observed by fluorescence microscopy (Olympus).

#### 2.1.5. Detection of Apoptotic Incidence by Flow Cytometry

After the application of cyclic stress and treatment under the abovementioned conditions, the cells were individually digested and collected using 0.05% trypsin-EDTA (Gibco). Subsequently, Annexin V/PI staining (C1062L, Beyotime) was performed according to the manufacturer's instructions. The cells were then assessed using flow cytometry. Annexin V+/PI− and Annexin V+/PI+ cells were considered as apoptotic cells in the early and late phases, respectively. The apoptotic cells were counted and expressed in terms of the percentage of the total cell count.

#### 2.1.6. Total Intracellular ROS Levels

Intracellular ROS production in primary cells was detected using a ROS assay kit (E004-1-1, Jiancheng Biochemical, Nanjing, China) according to the manufacturer's instructions. Briefly, following treatment, the cells (1 × 10^5^/mL) were treated with 10 *μ*M DCFH-DA—prepared in phosphate-buffered saline (PBS)—in the dark at 37°C for 30 min and subsequently washed with PBS to remove the excess dye. The fluorescence intensity was measured using a fluorescence spectrophotometer (Molecular Devices, Sunnyvale, CA, USA) at an excitation/emission wavelength of 490/585 nm.

#### 2.1.7. Measurement of SOD and MDA Levels and GPx Activity

After the cells (1 × 10^5^/mL) were treated as described above, the levels of SOD (A001-2-1) and MDA (A003-1) and the activity of GPx (H545-1-1) were measured using assay kits (Jiancheng Biochemical, Nanjing, China) according to the manufacturer's instructions.

#### 2.1.8. Detection of Changes in the Mitochondrial Membrane Potential via JC-1 Staining

Following treatment according to the abovementioned methods, cells were washed three times with PBS, stained with the JC-1 staining solution (C2006, Beyotime), and observed under a fluorescence microscope (Olympus). Green fluorescence (JC-1 monomers) indicated a decrease in the mitochondrial membrane potential and early apoptosis, whereas red fluorescence (JC-1 aggregates) indicated a relatively normal mitochondrial membrane potential and cell state. Hence, a decreased red/green fluorescence intensity ratio indicated decreased mitochondrial membrane potential.

#### 2.1.9. Detection of the Activities of Caspase-3, Caspase-8, and Caspase-9

After treatment according to the abovementioned methods, detection kits (Beyotime) were used to measure the activities of caspase-3 (C1115), caspase-8 (C1151), and caspase-9 (C1157) according to the manufacturer's instructions. Subsequently, the absorbance of each sample was measured at 405 nm on a microplate spectrophotometer (BioTek). The caspase activity in each well was expressed as the ratio of the A405-phase enzyme activity in each well to that of cells in the control group.

#### 2.1.10. Western Blot Analysis

After the application of cyclic stress and treatment according to the abovementioned methods, whole cell extracts were separated by 8% sodium dodecyl sulfate–polyacrylamide gel electrophoresis and electrotransferred to a polyvinylidene difluoride (PVDF) membrane (Bio-Rad, Hercules, CA, USA). The PVDF membrane was then incubated with 10 mM TBS with 1.0% Tween 20 and 10% dehydrated skim milk to block nonspecific protein binding. The membranes were subsequently incubated overnight with rabbit polyclonal antibodies against p-I*κ*B*α* (1 : 1000 dilution, ab133462), P65 (NF-*κ*B; 1 : 1000 dilution, ab19870), p-P65 (p-NF-*κ*B, 1 : 1000 dilution, ab194726) (Abcam, Cambridge, UK), cytochrome C (1 : 750 dilution, AF2047), and GAPDH (1 : 750 dilution, AF1186) (Beyotime) at 4°C. Subsequently, the membranes were washed with TBST and incubated with a horseradish peroxidase-conjugated secondary antibody (111-035-003; 1 : 1000 dilution, Jackson ImmunoResearch, PA) at 37°C for 1 h. The protein bands were visualized using an enhanced chemiluminescence reagent (Thermo Fisher Scientific, Waltham, MA, USA). Protein expression (normalized to that of GAPDH) was analyzed using the ImageJ software (National Institutes of Health, Bethesda, MD, USA).

### 2.2. *In Vivo* Experiments

#### 2.2.1. Preparation of an IVDD Rat Model and Drug Administration

Forty-eight 3-month-old male SD rats were randomly divided into four groups (*n* = 12 per group): IVDD group, sham group, low-concentration (100 mg/kg) naringin treatment group, and high-concentration (200 mg/kg) naringin treatment group. For establishing the IVDD rat model using static and dynamic imbalance, rats were anesthetized by intraperitoneally injecting ketamine (75 mg/kg), after which their limbs were fixed in the prone position, their skin was prepared, and disinfection and draping were performed. Next, the lower thoracic vertebrae, lumbar vertebrae, and upper sacrum were exposed using the posterior median approach. Once the erector spinae of the lumbar vertebrae, all lumbar spinous processes, supraspinous ligaments, interspinous ligaments, and posterior medial 1/2 of bilateral lumbar facet joints were removed, the wound was washed with normal saline containing 100 U/mL penicillin, and the deep fascia was closed to develop an unbalanced dynamic model with static forces of the lumbar spine [25]. Alternatively, in the sham group, only the skin on the dorsal surface was incised and sutured, and the rats were used as controls. One week after the operation, rats in the treatment groups were treated with naringin solution at high or low concentrations by intragastric administration. Naringin was diluted with normal saline to achieve a final concentration of 40 mg/mL. Owing to the slight solubility of naringin, fresh suspensions of naringin in normal saline were prepared each day. Before administration, the naringin suspension was shaken well with suspended magnetic stirring to ensure its homogeneity. Meanwhile, rats in the IVDD (vehicle group) and sham groups were intragastrically administered normal saline (6 mL/kg) daily.

#### 2.2.2. Magnetic Resonance Imaging (MRI) Examination

After 3 months of treatment, all rats were anesthetized by intraperitoneally injecting ketamine (75 mg/kg). MRI examinations were performed on all rats using a 3.0 T scanner (Siemens Symphony, Munich, Germany) with a dedicated custom-made animal volume resonator at room temperature. T2-weighted sections in the sagittal plane were obtained. The degree of IDD was estimated by two observers who were blinded to the study groups using the Pfirrmann MRI grade system (I–V) according to the distinction of NP (nucleus pulposus) and AF, structure, signal intensity, and height of the intervertebral disc [26].

#### 2.2.3. Determination of the Oxidative Stress Index in the Serum and Intervertebral Disc Tissues

Following MRI examination, serum samples were collected, and all rats were euthanized by the intraperitoneal overdose of pentobarbital sodium (150 mg/kg). Intervertebral disc tissue was collected from each group (four samples for oxidative stress analysis, four for histology staining, and four for immunohistochemistry). To evaluate the degree of oxidative stress, frozen intervertebral disc tissues were homogenized, and the supernatant was collected for analysis. The expression of ROS, SOD, GRx, and MDA in the serum and intervertebral disc was detected using the kits used for the *in vitro* experiments (Jiancheng Biochemical, Nanjing, China).

#### 2.2.4. Tissue Dissection

*(1) Hematoxylin and Eosin (H&E) Staining*. The rats were euthanized after surgery, and samples (L4/5 and L5/6 segments) were collected from each group, fixed with formaldehyde, decalcified, and embedded in paraffin. Subsequently, the specimens were sliced into 4 *μ*m sections. Following this, H&E staining was performed. The degree of degenerative changes in the AF was determined according to the criteria established by Nishimura and Mochida [27], as follows: Grade 1, mildly serpentine with rupture; Grade 2, moderately serpentine with rupture; Grade 3, severely serpentine with mildly reversed; Grade 4, severely reversed contour; or Grade 5, indistinct. The average value for the two segments was considered as the degeneration grade of the lumbar intervertebral discs in each rat.

*(2) TUNEL Staining*. TUNEL assay was performed on paraffin-embedded disc tissue sections using the one-step TUNEL apoptosis assay kit (Beyotime, C1089) according to the manufacturer's instructions. After Proteinase K treatment, samples were incubated with the TUNEL reaction mixture for 1 h at 37°C in the dark and then washed three times in PBS. Finally, the tissue sections were incubated with DAPI (Beyotime, C1002) for 10 min in the dark and then rewashed three times in PBS. The condensed or fragmented nuclei of apoptotic cells (red) were observed using fluorescence microscopy. The total apoptosis rate of the intervertebral disc cells from each rat was calculated as follows: (positive cells/total cells) × 100%.

#### 2.2.5. Immunohistochemistry-Based Detection of p-P65, MMP-3, and Type I Collagen Expression in the Intervertebral Disc Tissue

Immunohistochemistry for evaluating the expression of p-P65, type I collagen, and MMP-3 was performed according to the manufacturer's instructions. After antigen retrieval using 0.4% pepsin solution and blocking by goat serum, the sections were incubated with primary antibodies (p-P65, ab194726; collagen I, ab270993; MMP-3, ab52915; 1 : 200 dilution, Abcam) overnight at 4°C and then treated with secondary antibodies at 37°C for 30 min (A0208; 1 : 200 dilution, Beyotime) before color development with DAB, followed by counterstaining with hematoxylin. The immunohistochemistry samples were observed using a light microscope.

#### 2.2.6. Statistical Analysis

All measurements were expressed as mean ± standard deviation. Data were processed using the SPSS 18.0 software (SPSS, Inc., Chicago, IL, USA). ANOVA with subsequent Fisher's PLSD test was performed to determine the significance of differences in pairwise comparisons. Alternatively, the Mann–Whitney *U* test was performed for *in vivo* comparisons between the experimental and control groups. *P* < 0.05 was defined as the significance level.

## 3. Results

### 3.1. Effect of Naringin on the Activity of Rat AF Cells

**Figure 1:**
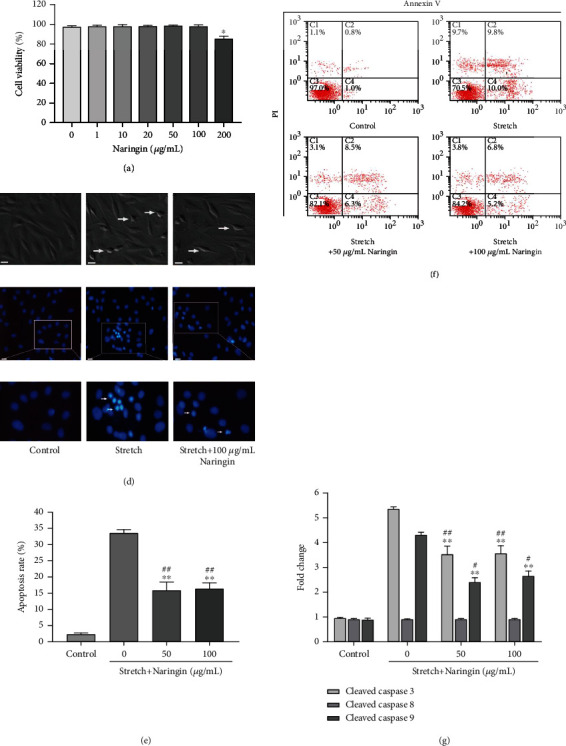
Effect of naringin on cyclic stretch-induced apoptosis in annulus fibrosus (AF) cells. (a) Effect of naringin on the cytotoxicity of rat AF cells. ^∗^*P* < 0.05 compared to the control group. (b) Effect of naringin based on morphological changes induced during rat AF cell apoptosis in response to cyclic stretch. Apoptotic cells were characterized by cell shrinkage and detachment from the silicone membrane (arrows). Scale bar: 100 *μ*m. (c, d) Morphological changes associated with apoptotic nuclei detected by Hoechst 33258 staining. Apoptotic nuclei showed the presence of condensed or fragmented DNA brightly stained with Hoechst 33258 (arrows). Scale bar: 50 *μ*m. (e) Effect of naringin on the apoptosis rate of rat AF cells stimulated by cyclic stretch. ^∗∗^*P* < 0.01 compared to the stretch group; ^##^*P* < 0.01 compared to the control group. (f) Representative graphs of flow cytometry analysis for cell apoptosis. (g) Effect of naringin on cyclic-stretch-induced caspase-3, caspase-8, and caspase-9 activation. ^∗∗^*P* < 0.01 compared to the stretch group. ^#^*P* < 0.05 and ^##^*P* < 0.01 compared to the control group.

We first investigated the toxicity of naringin toward rat AF cells following treatment with increasing concentrations for 48 h. MTT assay revealed that naringin did not exhibit significant toxicity toward rat AF cells at doses of up to 100 *μ*g/mL (Figure 1(a)). Therefore, 100 *μ*g/mL naringin was selected for subsequent experiments.

### 3.2. Naringin Ameliorates the Apoptosis of Rat AF Cells Induced by Cyclic Stretch

After 36 h of stretching, the cells gradually exhibited an apoptotic morphology, including cell rounding and shrinkage, vacuolization in the cytoplasm, shedding of smaller fragments from the cells, and detachment from the silicone membrane. Treatment with high and low concentrations of naringin inhibited these effects (Figure 1(b)). Hoechst 33258 staining showed a significant increase in chromatin condensation and nuclear fragmentation, which was effectively inhibited by naringin (Figures 1(c) and 1(d)). Flow cytometry analysis further revealed that the rate of apoptosis of rat AF cells in the control group was only 2.3 ± 0.8%, whereas the rate increased to 34.1 ± 1.6% in the stretch group. In addition, compared with that in the stretch group, the apoptosis rates significantly decreased in the high and low naringin concentration groups (Figures 1(e) and 1(f)).

### 3.3. Naringin Inhibits the Cyclic Stretch-Induced Activation of Caspase-3 and Caspase-9 in Rat AF Cells

The levels of the cleaved caspase-3 and caspase-9 in the stretch group increased significantly following cyclic stretch for 36 h. In contrast, compared to those in the stretch group, the cleaved caspase-3 and caspase-9 in the high- and low-concentration naringin treatment groups were significantly inhibited (Figure 1(g)). However, the level of the cleaved caspase-8 did not differ between the groups.

### 3.4. Naringin Inhibits Cyclic Stretch-Induced Mitochondrial Membrane Potential Depolarization and Cytochrome C Release

**Figure 2:**
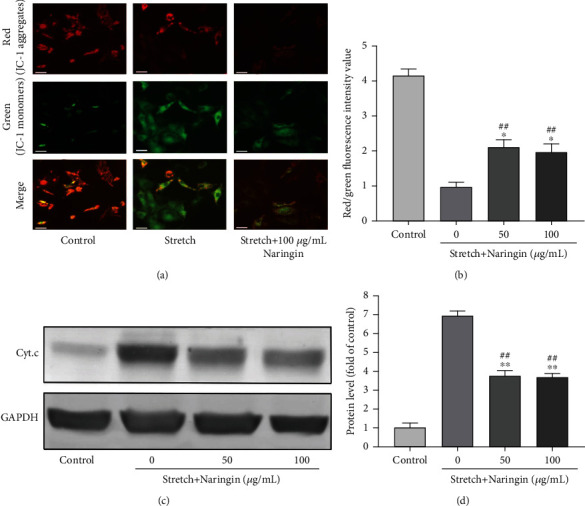
Effect of naringin on mitochondrial damage induced by cyclic stretch. (a) Representative images of cells stained with JC-1 after cyclic stretch for 36 h. Original magnification: ×200. Scale bar: 50 *μ*m. (b) Ratio of red/green fluorescence intensity used to determine the mitochondrial membrane potential and perform semiquantitative analysis. (c) Representative western blot for the detection of cytoplasmic cytochrome C. (d) Semiquantitative analysis of cytoplasmic cytochrome C levels. ^∗^*P* < 0.05 and ^∗∗^*P* < 0.01 compared to the stretch group; ^##^*P* < 0.01 compared to the control group.

After cyclic stretch was applied for 36 h, the ratio of red (JC-1 aggregates) to green (JC-1 monomers) fluorescence intensities decreased, indicating that cyclic stretch suppressed the mitochondrial membrane potential. Compared with that in the stretch group, the mitochondrial membrane potential increased significantly in the high- and low-concentration naringin groups, but did not increase to the level in the control group. Our results indicate that naringin inhibited mitochondrial membrane potential depolarization induced by cyclic stretch in AF cells (Figures 2(a) and 2(b)). In addition, the cytoplasmic levels of cytochrome C increased substantially after cyclic stretch for 36 h, but naringin significantly inhibited the release of cytochrome C into the cytoplasm in the high- and low-concentration naringin treatment groups (Figures 2(c) and 2(d)).

### 3.5. Naringin Inhibits Cyclic Stretch-Induced Oxidative Stress

**Figure 3:**
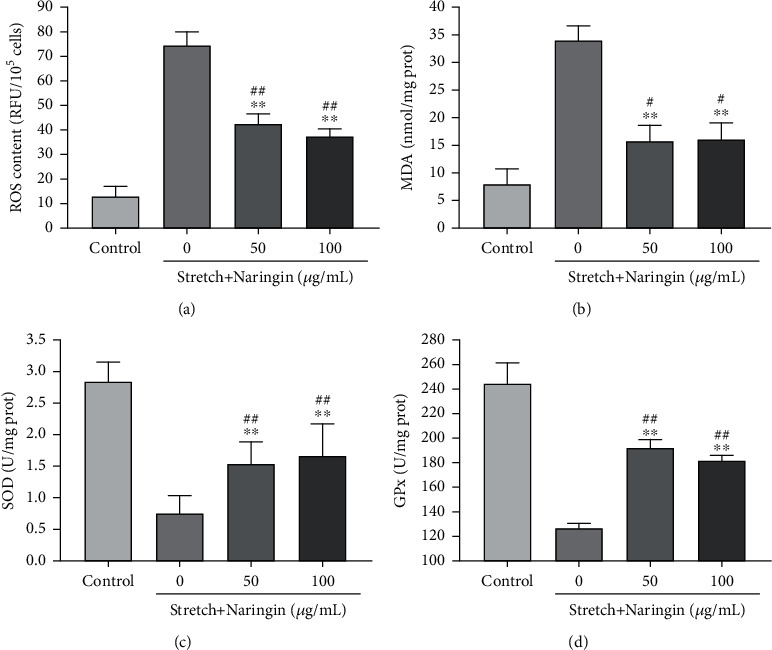
Effect of naringin on oxidative stress induced by cyclic stretch in AF cells (a–d). Naringin inhibited reactive oxygen species (ROS) production, reduced the malondialdehyde (MDA) level, and increased the superoxide dismutase (SOD) and glutathione peroxidase (GPx) levels. ^∗∗^*P* < 0.01 compared to the stretch group. ^#^*P* < 0.05 and ^##^*P* < 0.01 compared to the control group.

Compared with that in the control group, a significant increase in ROS and MDA levels and a significant decrease in SOD and GPx levels were observed in the stretch group. Alternatively, compared with that in the stretch group, the levels of ROS and MDA in the high- and low-concentration naringin treatment groups decreased significantly, whereas the levels of SOD and GPx increased significantly. However, no significant differences were observed between the levels of the different components in the high- and low-concentration groups (Figure 3). Our results indicate that cyclic stretch-induced oxidative stress in AF cells could be inhibited by naringin.

### 3.6. Naringin Inhibits the Cyclic Stretch-Induced Activation of the NF-*κ*B Signaling Pathway

**Figure 4:**
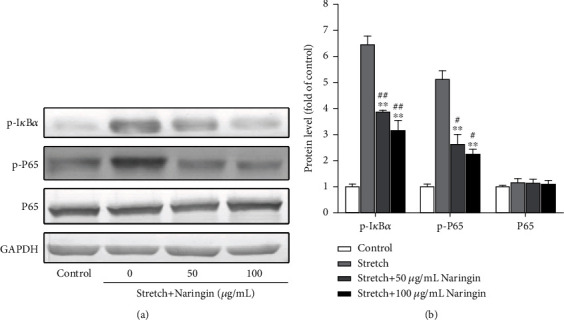
Effect of naringin on the levels of NF-*κ*B pathway markers (proteins). (a) Representative western blots showing the effect of naringin on the expression of p-I*κ*B*α*, p-P65, and P65 proteins after cyclic stretch exposure for 36 h; (b) semiquantitative analysis of p-I*κ*B*α*, p-P65, and P65 expression. ^∗∗^*P* < 0.01 compared to the stretch group. ^#^*P* < 0.01 and ^##^*P* < 0.01 compared to the control group.

The expression of p-I*κ*B*α* and p-P65 increased significantly in the stretch group compared to that in the control group. Compared with that in the stretch group, the expression of p-I*κ*B*α* and p-P65 proteins in the high- and low-concentration naringin treatment groups decreased significantly; however, there was no significant difference between the levels in the high- and low-concentration groups. In addition, no significant changes were observed in the total P65 expression between the groups. Our results indicate that naringin inhibited the cyclic stretch-induced activation of the I*κ*B*α*/NF-*κ*B pathway (Figures 4(a) and 4(b)).

### 3.7. Naringin Delays Disc Degeneration, as Observed by MRI

**Figure 5:**
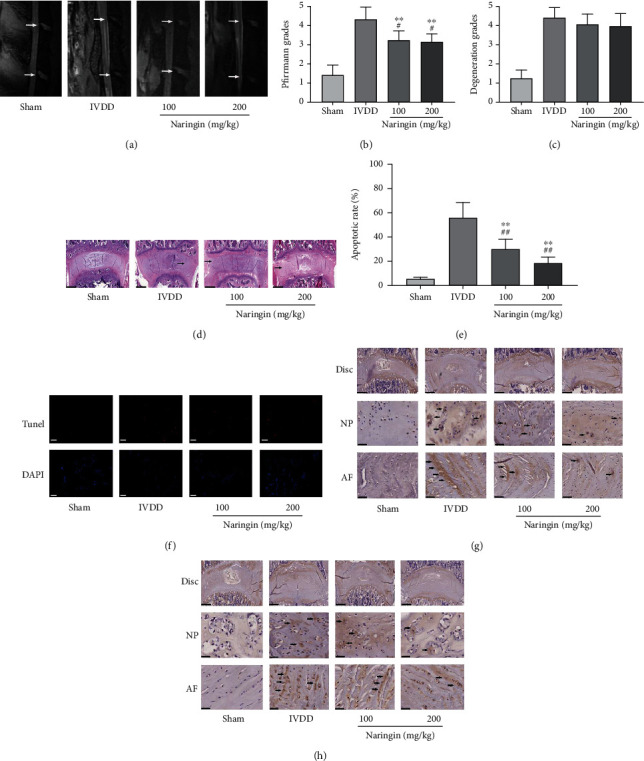
Effect of naringin on rat lumbar intervertebral disc degeneration (IVDD). (a) Representative sagittal MRI scans of the rat lumbar spine. Degenerative discs show the lost distinction of NP and AF, decreased collapsed disc space, hypointense on T2 images (“black holes”). Intervertebral discs are marked by arrows. (b) Pfirrmann grade indicating the degree of IVDD in the rat study groups. ^∗∗^*P* < 0.01 compared to the sham group. ^#^*P* < 0.05 compared to the IVDD group. (c, d) Representative hematoxylin and eosin staining images and degeneration grade in the intervertebral discs. Degenerative discs showed moderately to severely serpentine appearance with rupture or reversed contour or were indistinct (arrows). Scale bar: 1 mm. (e, f) Typical TUNEL staining (red) images of the rat lumbar spine demonstrating the apoptosis rate. ^∗∗^*P* < 0.01 compared to the sham group. ^##^*P* < 0.01 compared to the IVDD group. Scale bar: 50 *μ*m. (g) Type I collagen expression in the intervertebral disc of rats from the naringin treatment groups increased compared with that of rats from the sham group, but decreased compared with that of rats from the IVDD group. Type I collagen-positive staining was shown in brown in ECM (arrows). Scale bar: 1 mm (disc) and 200 *μ*m (NP and AF). (h) MMP-3 expression in the intervertebral disc of rats from the naringin treatment groups increased compared with that of rats from the sham group, but decreased compared with that of rats from the IVDD group. MMP-3-positive staining was shown in brown in ECM (arrows). Scale bar: 1 mm (disc) and 200 *μ*m (NP and AF). AF: annulus fibrosus; NP: nucleus pulposus; ECM: extracellular matrix.

MRI revealed normal intervertebral discs in the sham group (stronger signal intensities on T2 images). After 3 months, the MRI of the discs in the high- and low-concentration naringin treatment groups showed stronger signal intensities than those in the IVDD group, which showed weaker signals (on T2 images) or “black holes.” In addition, the degree of disc degeneration, evaluated using the Pfirrmann grade, was significantly lower (better) in the high- and low-concentration naringin treatment groups compared to the IVDD group, although no significant differences were observed between the two treatment groups (Figures 5(a) and 5(b)).

### 3.8. Naringin Marginally Decreases IVDD Grading, as Observed by H&E Staining

After 3 months, the discs in the control group showed a normal or mildly serpentine appearance, whereas those in the IVDD group and the two naringin treatment groups showed mild to moderate serpentine appearances with reversed contour, rupture, or indistinct structures. Meanwhile, compared with that in the IVDD group, the degree of degeneration in the high- and low-concentration naringin treatment groups decreased slightly, although the difference was not significant (Figures 5(c) and 5(d)).

### 3.9. Naringin Inhibits AF Cell Apoptosis In Situ

At 3 months after treatment, fewer apoptotic cells were visible within the discs of control rats compared to the other three groups. Meanwhile, the apoptosis rates in the high- and low-concentration naringin treatment groups were significantly lower than that in the IVDD group (Figures 5(e) and 5(f)).

### 3.10. Naringin Attenuates the Increase in Type I Collagen and MMP-3 Expression *In Situ*

The increase in type I collagen expression was an indicator of IVDD. Immunohistochemistry revealed that the staining for type I collagen in the IVDD group increased compared to that in the sham group. The expression of type I collagen in the high- and low-concentration naringin treatment groups was lower than that in the IVDD group. As a catabolic enzyme, MMP-3 showed an expression trend similar to that of type I collagen. (Figures 5(g) and 5(h)).

### 3.11. Naringin Inhibits Oxidative Stress in IVDD Rat Serum and Intervertebral Disc Tissues

**Figure 6:**
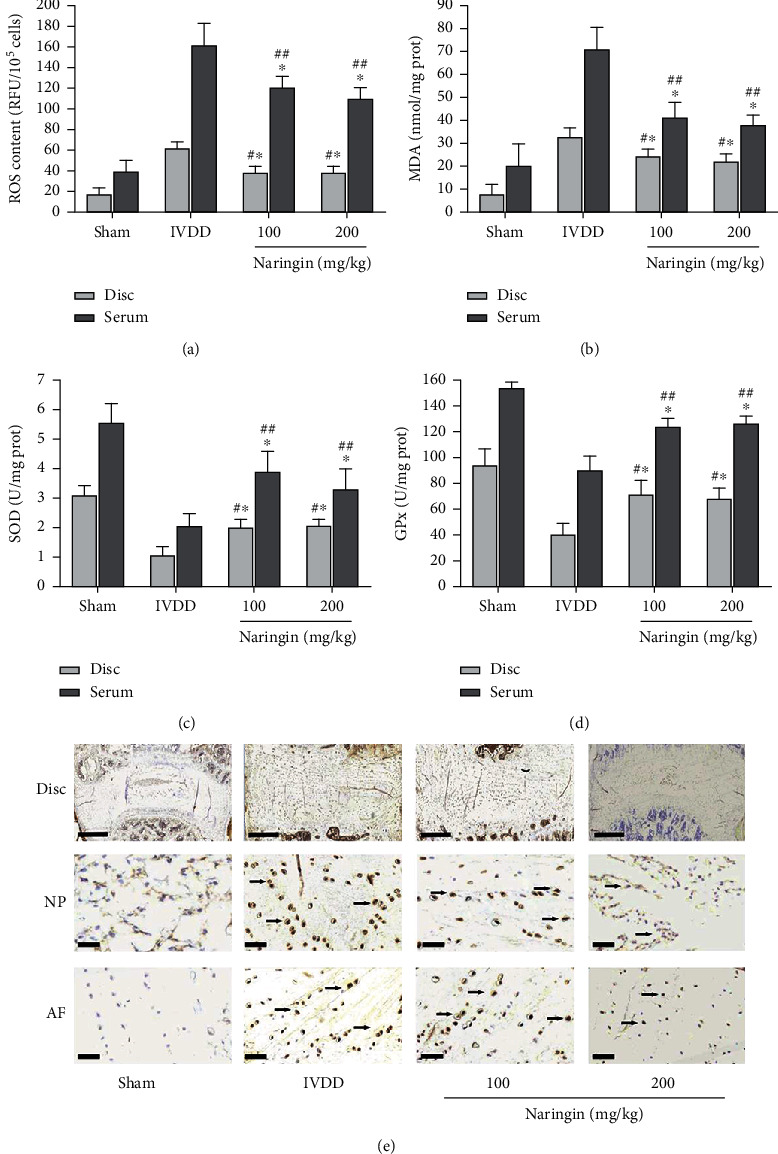
Effect of naringin on oxidative stress and p-P65 expression in rat lumbar interventricular disc degeneration (IVDD) induced by unbalanced dynamic and static forces. (a–d) Effect of naringin on oxidative stress in serum and intervertebral disc tissues: (a) reactive oxygen species (ROS) production, (b) malondialdehyde (MDA), (c) superoxide dismutase (SOD), and (d) glutathione peroxidase (GPx) levels. ^∗^*P* < 0.05 and ^∗∗^*P* < 0.01 compared to the sham group. ^#^*P* < 0.05 and ^##^*P* < 0.01 compared to the IVDD group. (e) Effect of naringin on p-P65 expression in the intervertebral discs, as observed by immunohistochemistry. The expression of p-P65 in the high- and low-concentration naringin treatment groups was lower than that in the IVDD group. The p-P65-positive cells are shown in brown (arrows). Scale bar: 1 mm (disc) and 200 *μ*m (NP and AF).

Compared with those in the sham group, the ROS and MDA levels in the serum and intervertebral discs increased, and the SOD and GPx activities decreased significantly, in the high- and low-concentration naringin treatment groups. However, compared with those in the IVDD group, the ROS and MDA levels decreased significantly, and the SOD and GPx activities increased considerably, in the high- and low-concentration naringin treatment groups. These results revealed that naringin could reduce the levels of oxidative stress caused by static and dynamic imbalance in rats (Figures 6(a)–6(d)).

### 3.12. Naringin Inhibits p-P65 Expression In Situ

Immunohistochemistry revealed that the expression of p-P65 in the high- and low-concentration naringin treatment groups was significantly lower than that in the IVDD group. However, no significant differences were observed between the two treatment groups (Figure 6(e)).

## 4. Discussion

Degenerative disc disease is associated with clinical syndromes that manifest as neck, shoulder, and leg pain, which can seriously affect the quality of life of affected individuals. At present, some patients with IVDD-related diseases undergo surgery to relieve compression and alleviate symptoms, even though such procedures may not prevent IVDD progression. Hence, researchers have attempted to develop drugs to alleviate or delay IVDD [28]. Naringin, a monomer extracted from *Rhizoma Drynariae*, has exhibited antioxidant, angiogenic, and anti-inflammatory properties, which make it a suitable candidate for the treatment of multiple degenerative diseases [17, 29]. Herein, we showed that naringin can reduce cyclic stretch-induced apoptosis and delay IVDD by inhibiting oxidative stress and the activation of the NF-*κ*B signaling pathway. Naringin may thus be effective for treating degenerative disc disease.

The intervertebral disc is the most critical part of the human spinal load-bearing system and has important functions, such as absorbing vibrations, relieving shock, and uniformly distributing external force. Intervertebral discs are tissues that undergo degenerative changes at an earlier time point compared to other body parts. Specifically, apoptosis can cause changes in the biomechanical properties of the ECM, thereby playing a vital role in the pathogenesis of degenerative disc disease [3]. Previous studies have confirmed that mechanical factors play a critical role in IVDD [28, 30]. We previously showed that cyclic stretch causes apoptosis in AF cells through the mitochondrial and endoplasmic reticulum pathways [5, 6]. Meanwhile, naringin has been suggested to inhibit the apoptosis of intervertebral disc cells, regulate cell metabolism, and inhibit IVDD [19, 22, 23]. In this study, naringin did not exert significant toxic effects on rat AF cells, and the apoptosis of rat AF cells was successfully induced by excessive cyclic stretch, similar to our previously reported findings [5, 6]. However, the rates of apoptosis in the high- and low-concentration naringin treatment groups decreased significantly compared to that in the stretch group, whereas it remained higher than those in the control group. These results indicate that even though naringin reduced the rate of cyclic stretch-induced apoptosis in AF cells, it failed to completely inhibit apoptosis.

ROS are unstable and highly active compounds, with or without free radicals, formed as byproducts of cellular oxidative metabolism [31]. The scavenging ability of intracellular antioxidants is insufficient when ROS are present in excess, and under such conditions, ROS cause oxidative damage to DNA, lipids, and proteins, which eventually lead to cell damage and death [32]. Therefore, ROS levels are related to the pathogenesis of several diseases, including cardiovascular diseases and osteoarthritis [33, 34]. Indeed, excess ROS are produced in degenerated intervertebral disc tissues, and the development and progression of IVDD is closely associated with ROS and oxidative stress [22, 35–37]. SOD and GPx are important free radical scavengers that serve as the first line of defense against oxidative damage and play important roles in protecting cells from oxidative damage. Alternatively, the level of MDA (the end product of lipid peroxidation) influences the degree of lipid peroxidation [7]. Research has shown that the GPx concentration decreased and the MDA concentration increased in human serum and intervertebral disc tissues under IVDD, and these indirectly reflected the degree of IVDD [38]. Additionally, naringin can inhibit the release of pain mediators by inhibiting the oxidative stress-induced apoptosis of nucleus pulposus cells and mesenchymal stem cells [21–23]. Our results confirmed that naringin can inhibit the cyclic stretch-induced increase in ROS and MDA levels in rat AF cells and enhance the expression of SOD and GPx, suggesting that the protective effect of naringin on the apoptosis of rat AF cells is related to its antioxidant activity.

The mitochondria are the primary targets of ROS. The permeability of the mitochondrial membrane increases after oxidative damage, which decreases the mitochondrial transmembrane potential and suppresses the release of cytochrome C into the cytoplasm [39]. Activated caspase-9 can induce apoptosis through the mitochondrial pathway, whereas apoptotic events in several pathways are predominantly mediated by cleaved caspase-3 [40]. Abnormal simulations, including high oxygen and glucose levels, and excessive mechanical stress can increase mitochondrial permeability, reduce membrane potential, and induce abnormal mitochondrial functions, thereby affecting the apoptosis of intervertebral disc cells and ECM metabolism, which subsequently influences the development of IVDD [41–43]. The activation of caspase-8 leads to apoptosis through the death receptor pathway. In the present study, consistent with findings from our previous study [5], cyclic stretch was found to exert no effect on caspase-8 activity, which suggested that the death receptor pathway was not involved in the cyclic stretch-induced apoptosis of AF cells. Our results revealed that cyclic stretch induced a significant increase in the expression of cytochrome C, cleaved caspase-3, and caspase-9, while decreasing the mitochondrial membrane potential, all of which relate to the apoptosis rate. These results suggest that cyclic stretch may induce apoptosis through the mitochondrial pathway. Our results indicate that naringin inhibits ROS production and enhances the antioxidant capacity of rat AF cells. This finding preliminarily indicates that naringin may inhibit the cyclic stretch-induced apoptosis of rat AF cells by suppressing oxidative stress and improving mitochondrial functions.

NF-*κ*B (P65) is a key transcription factor. In the resting state, P65 expression is restricted to the cytoplasm via I*κ*B inhibition [44]. Following stimulation by products of oxidative stress, such as ROS, I*κ*B is phosphorylated, which leads to P65 activation. Activated P65 is phosphorylated and translocated to the nucleus to regulate the release of related inflammatory cytokines, increase the expression of inflammatory factors, and aggravate the inflammatory response [45, 46]. Moreover, P65 and ROS exhibit mutual stimulation to enter a vicious circle, further aggravating cell damage [10, 45]. Our results revealed that cyclic stretch stimulated rat AF cells, thereby promoting the phosphorylation of I*κ*B*α* and P65, decreasing the mitochondrial membrane potential, and promoting the overexpression of cleaved caspase-3 and caspase-9, which indicated that cyclic stretch activates the NF-*κ*B pathway and causes apoptosis. Naringin can inhibit the phosphorylation of I*κ*B*α* and P65, thereby antagonizing cyclic stretch-induced apoptosis.

We further verified the effect of naringin on IVDD and the underlying mechanism using a rat model of IVDD induced by dynamic and static imbalance. In this model, most ligaments and major muscles at the back of the lumbar spine were removed, which inevitably destabilized the lumbar spine, such that the AF bore a greater tensile load during the regular activities of the rats. Moreover, this did not directly damage the intervertebral disc tissue and accurately reflected the pathogenesis of IVDD [25, 47]. There is evidence that naringin has a good safety profile and is nontoxic to rats even at doses greater than 1250 mg/kg/day when administered by oral gavage for 13 consecutive weeks or 6 consecutive months [48, 49]. Similarly, a recent study performed using a rat model of puncture-induced IVDD treated with a 80 mg/kg/day dose of naringin revealed that naringin protects nucleus pulposus cells against apoptosis and ameliorates IVDD *in vivo* [21]. Moreover, in our previous study, in which we used an ovariectomized rat model of osteoporosis treated with 100 or 200 mg/kg/day naringin, we observed that naringin exerted its antiosteoporotic effect by regulating endothelial cell functions and promoting angiogenesis [50]. Based on these results, the doses of 100 and 200 mg/kg were adopted for the *in vivo* investigations performed in the current study. At these doses, compared with those in the IVDD group, the intervertebral discs of rats in the treatment groups exhibited relatively normal T2 hyperintense signals on MRI, significantly low TUNEL-positive cells counts, and low p-P65 expression. Furthermore, the ROS and MDA levels decreased in the sera and intervertebral discs of rats in the high- and low-concentration naringin treatment groups, whereas the SOD and GPx expression increased. Our *in vivo* experiments further demonstrated that naringin inhibited the activation of the NF-*κ*B pathway, reduced the oxidative stress response, and inhibited intervertebral disc cell apoptosis, thereby delaying the degeneration of intervertebral discs. In addition, the expression of type I collagen and MMP-3 in the high- and low-concentration naringin treatment groups was significantly lower than that in the IVDD group. Meanwhile, H&E staining revealed no significant differences in the IVDD score, which is likely attributable to the fact that type I collagen staining is more sensitive than H&E staining for IVDD detection. Moreover, the treatment time may have been insufficient for demonstrating a delayed IVDD effect.

With respect to the method of administration, intradiscal injection can be used to rapidly achieve a high naringin concentration in local disc cells; however, previous studies have confirmed that needle puncture can directly cause IVDD [51, 52]. Moreover, the disc is virtually avascular. The transport of nutrients in the disc primarily depends on diffusion from the capillary beds of the cartilaginous endplate (the predominant route) and the peripheral annulus. The metabolic products are removed by the reverse route. Therefore, naringin was administered via the intragastric route in the present study. Li et al. [48, 49] investigated the potential systemic effects of the oral administration of naringin and showed that naringin is practically nontoxic in rats when administered orally for 13 consecutive weeks and even for 6 months. Similarly, naringin administered orally for 3 and 6 consecutive months at a minimum dose of 500 mg/kg body weight per day showed a good safety profile in beagles [53]. In view of the results of previous experiments, we did not evaluate the potential systemic effects of naringin in this study. However, these effects should be investigated before the clinical application of naringin.

This study had certain limitations. First, as we primarily focused on the antioxidant effects of naringin on rat AF cells, it remains unclear whether the phenotype of rat nucleus pulposus cells can be regulated by naringin, and this warrants further investigation. Second, although previous studies have shown that the effects of naringin are time- and dose-dependent [19, 21–23], only two concentration groups were used in the current study; hence, further animal and clinical studies are required to optimize the dose and duration of naringin treatment for IVDD. Lastly, the animal model used in the present study does not accurately reflect the disease pathology in humans, as rats and humans differ in multiple aspects, such as cell phenotypes, discs, spinal anatomy, and mechanical properties of cells [54]. Nevertheless, the use of an animal model has helped advance our understanding of disc degeneration induced by excessive stretch load.

## 5. Conclusions

Naringin can reduce AF cell apoptosis induced by cyclic stretch and delay IVDD by inhibiting oxidative stress and activation of the NF-*κ*B signaling pathway. Therefore, it may serve as a potential therapeutic agent for IVDD. However, as naringin has several pharmacological properties, its systemic effects on other tissues and organs should be explored before its clinical application.

## Data Availability

Data is available upon request from corresponding authors.
